# *Euonymus saxatilis* (Celastraceae), a new species from karst regions in Southwest China

**DOI:** 10.3897/phytokeys.280.195001

**Published:** 2026-10-22

**Authors:** Jian Qiu, Xu Wu, Ming-Tai An, Jiang-Hong Yu, Guang-Lin Li

**Affiliations:** 1 College of Forestry, Guizhou University, Guiyang 550025, Guizhou, China College of Forestry, Guizhou University Guiyang China; 2 Key Laboratory of Plant Resource Conservation and Germplasm Innovation in Mountainous Region (Ministry of Education), College of Life Sciences/Institute of Agro-bioengineering, Guizhou University, Guiyang 550025, Guizhou, China Key Laboratory of Plant Resource Conservation and Germplasm Innovation in Mountainous Region (Ministry of Education), College of Life Sciences/Institute of Agro-bioengineering, Guizhou University Guiyang China

**Keywords:** Karst, molecular phylogeny, morphological comparison, new taxon

## Abstract

A new species *Euonymus saxatilis* M.T.An, Jian Qiu & Xu Wu, **sp. nov**. (Celastraceae) from Guangxi Zhuang Autonomous Region, China, is described and illustrated based on morphological characteristics and molecular evidence. Morphologically, the species is most similar to *E. dielsianus*, but differs in the following characters: leaves obovate or obcordate, inflorescence 1–3-flowered, peduncle 2–4.5 cm, style distinct. Phylogenetic analyses based on ITS and *trnL-F* sequences strongly support the status of *E. saxatilis* as a distinct new species within the genus *Euonymus* (BS = 100%), which is resolved as sister to *E. dielsianus* with strong bootstrap support (BS = 98%).

## Introduction

The genus *Euonymus* L. belongs to Celastraceae and comprises approximately 220 species in the world (Li et al. 2022). It originated in Europe and most of its species are distributed in tropical and temperate regions of the Northern Hemisphere, whereas only a few have radiated into the cold temperate zone (Ma 2001; Qin et al. 2025). The diversity of this genus is mainly concentrated in southwestern China, South Asia, and adjacent Himalayan countries, with more than half of the species occurring in China (Li et al. 2024). Members of the genus are shrubs or small trees, characterized by opposite or sub-opposite leaves (rarely alternate or 3-whorled), axillary thyrses, 4–5-merous flowers, stamens inserted on the disc periphery, loculicidal dehiscent capsules and arillate seeds.

The genus *Euonymus* shows high morphological variability, and its taxonomy has long been debated (Li et al. 2014). Fruit characters serve as the core basis for its infrageneric classification in traditional taxonomic systems. Blume (1827) was the first to use fruit characters for the taxonomy of the genus. He divided *Euonymus* into two sections based on fruit morphology, which has been crucial for subsequent taxonomic efforts. Not until 1902 did scholars begin to focus on the diverse morphological characters of vegetative organs, rather than relying solely on floral and fruit traits (Loesener 1902). In the Flora Reipublicae Popularis Sinicae (FRPS), Cheng et al. (1999) subdivided the genus into five sections based on capsule morphology: sect*. Euonymus*, sect. *Echinococcus* Nakai, sect. *Ilicifolii* Nakai, sect. *Melanocarya* (Turcz.) Nakai and sect. *Uniloculares* Rouy & Foucaud. The sections are mainly distinguished by fruit characters: *E.* sect. *Uniloculares* has winged capsules and unilocular anthers; *E.* sect. *Melanocarya* possesses capsules 4-lobed almost to the base; *E.* sect*. Echinococcus* bears capsules armed with spines or tubercles; *E.* sect*. Ilicifolii* has smooth, suborbicular or globose capsules; *E.* sect. *Euonymus* usually features rugose capsules with costae. This classification system based on capsule morphology has been widely adopted and applied in subsequent taxonomic and floristic studies of the genus *Euonymus* in China (Du et al. 2015).

With advances in molecular phylogenetic studies, traditional taxonomic systems often do not fully correspond to the true evolutionary relationships. The genus *Euonymus* was considered to be closely related to *Glyptopetalum*. The taxonomy of *Glyptopetalum* has long remained controversial. Some taxonomists treated it as a distinct genus based on morphological evidence (Hou 1963; Meng et al. 2011; Simmons et al. 2012), whereas others either omitted mention of this genus or recognized it as an infrageneric taxon within *Euonymus* (Hu et al. 2022). With the continuous progress of research, numerous new species have been formally described and published, such as *E. aquifolium* (Hu et al. 2022), *E. chengduanus* (Hu et al. 2025), *E. maotaiensis* (Wu et al. 2026). In all these studies, *Glyptopetalum* was found nested within the *Euonymus* clade. Based on a numerical taxonomic analysis, Liu et al. (2021) further supported the reduction of *Glyptopetalum* to a section within *Euonymus*. Accordingly, this study adopts a broad generic circumscription of *Euonymus*, treating *Glyptopetalum* as *E.* sect. *Glyptopetalum*. This generic taxonomic revision will affect the infrageneric classification of *Euonymus*, challenging the traditional practice of delimiting infrageneric taxa solely by fruit characters. Therefore, future studies should incorporate a broader suite of morphological characters combined with multi-locus molecular markers to further refine the existing sectional classification of *Euonymus*.

Mulun National Nature Reserve is located in the northwest of Huanjiang Maonan Autonomous County, Guangxi Zhuang Autonomous Region, China (Liu et al. 2012). Located on the southern edge of the mid-subtropical zone, the region features a mid-subtropical humid monsoon climate, which provides favorable natural conditions for plant growth and reproduction (Tan et al. 2023). During the field survey conducted in July 2025, several individuals of the genus *Euonymus* were discovered at an elevation of approximately 750 m in the reserve. These specimens exhibited distinct morphological characters that clearly separated them from all previously described species of the genus *Euonymus* species. Subsequently, we carried out detailed morphological comparisons with their closely related taxa, and verified that this entity differs significantly from all other congeneric species in multiple diagnostic morphological characters.

## Materials and methods

### Morphological observations

Morphological characteristics of the new species were obtained from observations and measurements of wild plants. We conducted comparative studies between the new species and its putative allies. All morphological characters were carefully examined using both field-collected and herbarium specimens under a binocular microscope. We examined specimens of closely related species via platforms including the Flora of China (FOC, https://www.iplant.cn/foc), the Chinese Virtual Herbarium (CVH, https://www.cvh.ac.cn/), the Global Biodiversity Information Facility (GBIF, https://www.gbif.org/), and the World Flora Online (WFO, https://www.worldfloraonline.org/). When *Euonymus dielsianus* Loes. & Diels was confirmed as the morphologically closest species, a thorough comparison with its type specimen was conducted. The comparison focused on key diagnostic traits including leaf shape, leaf margin, inflorescence architecture, pedicel length, flower diameter, style morphology, and stamen characteristics. Voucher specimens are deposited in the GZAC herbarium.

### DNA extraction and sequencing

In this study, a total of 61 accessions representing 55 species from 3 genera of Celastraceae were included in the phylogenetic analysis. The genera *Tripterygium* Hook.f. and *Celastrus* L. were chosen as the outgroups. With the exception of the newly generated sequences for *E. saxatilis* and *E. maotaiensis*, all other nucleotide sequences used in the molecular phylogenetic analyses were retrieved from the NCBI GenBank. The NCBI GenBank accession numbers for the material used in this study are provided in Appendix 1.

We collected fresh green leaves of the new species from Guangxi, China, and immediately preserved them in silica gel for subsequent DNA extraction. Total genomic DNA was extracted from silica-dried leaves using a modified CTAB method (Murray and Thompson 1980). We sequenced the nuclear ribosomal internal transcribed spacer (ITS) and the chloroplast intergenic spacer *trnL-F*. The ITS region was selected as the molecular marker for phylogenetic analyses and amplified via PCR with the universal primer pair ITS1/ITS4 (White et al. 1990). The chloroplast *trnL-F* fragment was amplified using its corresponding specific primers. All samples were sent to Sangon Biotech for sequencing. The resulting sequences of *E. saxatilis* and *E. maotaiensis* have been deposited in the NCBI GenBank database, with their accession numbers detailed in Appendix 1.

### Phylogenetic analyses

To reveal the phylogenetic relationships of *E. saxatilis* and its related species, we newly generated ITS and *trnL-F* sequences for *E. saxatilis* and combined published sequence data from previous phylogenetic studies to reconstruct a multi-locus concatenated phylogenetic tree. A total of 61 sequences representing 55 species were included in the analysis. Data processing and analyses were conducted in PhyloSuite v1.2.3 (Zhang et al. 2020). All sequences were aligned using MAFFT v.7.475 with default parameters (Katoh and Standley 2013). Prior to sequence concatenation, ambiguous and low-quality alignment sites were trimmed using trimAl (Capella-Gutierrez et al. 2009). The best-fit nucleotide substitution model was selected via the built-in ModelFinder (Kalyaanamoorthy et al. 2017). Based on the aligned sequences, maximum likelihood (ML) analysis was performed using IQ-TREE (Nguyen et al. 2015). Bootstrap analyses were performed with 1,000 replicates. ModelFinder selected the best-fit partition scheme and nucleotide substitution models based on the Bayesian Information Criterion (BIC). The GTR+F+I+G4 model was assigned to the ITS partition, and the K3Pu+F+G4 model was assigned to the *trnL-F* partition.

## Taxonomic treatment

### Euonymus saxatilis

Taxon classificationPlantaeCelastralesCelastraceae

M.T.An, Jian Qiu & Xu Wu sp. nov.

EB9D4A58-C7D8-5F75-A58E-BF6E4155DD72

urn:lsid:ipni.org:names:77422914-1

Figs 2, 3

#### Diagnosis.

Morphologically, *E. saxatilis* is similar to *E. dielsianus*, but can be distinguished by the following key characters (Table 1): leaves obovate or obcordate (vs. obovate or elliptic-obovate), margin sparsely shallowly crenulate-serrulate for more than 2/3 and entire for the remaining portion (vs. proximal 1/5–2/5 entire, distal 3/5–4/5 crenulate), inflorescence 1–3-flowered (vs. 1–7-flowered), flower diameter 1.5 cm (vs. ca. 0.6 cm), styles distinct (vs. style absent).

**Table 1.** Morphological comparisons of *Euonymus saxatilis* and *E. dielsianus*.

**Characters**	***E. saxatilis***	***E. dielsianus***
Leaf texture	Thickly coriaceous	Coriaceous or thickly coriaceous
Leaf shape	Obovate or obcordate	Obovate or elliptic-obovate
Leaf margin	Upper part sparsely serrate, elsewhere entire	Proximal 1/5–2/5 entire, distal 3/5–4/5 crenulate
Leaf size	5–12 × 1.5–6 cm	9–15 × 4.5–6 cm
Petiole length	0.5–1.5 cm	Ca. 1 cm
Inflorescence	1–3-flowered	1–7-flowered
Peduncle	2–4.5 cm	1.5 cm
Flower diameter	1.2–1.6 cm	Ca. 0.6 cm
Petals	Suborbicular	Ovate
Style	Distinct, 1.5 mm long	Absent
Filament	Distinct, ca. 1.5 mm	Very short
Capsule	4-lobed to the base	Deeply 4-lobed

#### Type.

China, Guangxi Zhuang Autonomous Region: Hechi City, Huanjiang Maonan Autonomous County, Chuanshan Town, 25°06'N, 107°57'E, alt. 750 m, 5 July 2025, *Ming-tai An, Xu Wu, Jian Qiu GZAC-WX-2575* (holotype: GZAC!; isotype: GZAC!).

#### Etymology.

The specific epithet is derived from the species’ habitat, which is a typical karst landform.

#### Vernacular name.

Chinese Mandarin: Yán shēng wèi máo (岩生卫矛).

#### Description.

Evergreen shrubs or small trees, 1–6 m tall; twigs terete, bark gray-brown, irregular longitudinal fissures; young branches green, old branches pale greyish brown. Leaves opposite, thickly coriaceous, obovate or obcordate, 5–12 cm × 1.5–6 cm, apex obtuse, acute or emarginate, base cuneate, margin sparsely shallowly crenulate-serrulate for more than 2/3, and entire for the remaining portion; veins prominent, midrib slightly convex adaxially, distinctly prominent abaxially, lateral veins 4–6 pairs; petioles 0.5–1.5 cm long. Cymes with 1–3 flowers, peduncle 2–4.5 cm long; pedicels up to 1.5 cm long; flowers 4-merous, ca. 1.5 cm in diam., greenish yellow; sepals broadly orbicular, margin white membranous; petals suborbicular, margin slightly revolute, irregularly incised; disk subquadrate; filaments distinct, ca. 1.5 mm long, inserted on margin of cupuliform disk, anthers 2-locellate, pale yellow, connate by two valves; ovary 4-angled; style distinct, dark green; stigma rounded. Capsule 4-lobed to the base, lobes ovoid, ca. 1 cm in diam., ascending, 1–3 lobes fertile, each with 1 mature seed; seeds ellipsoid, ca. 5 mm long, red or orange-red; the seed spine without branches near the seed confluence; aril helmet-shaped, enclosing the upper half of the seed.

#### Phenology.

Flowering from June to July, and fruiting from September to November.

#### Distribution and habitat.

**Figure 1.**
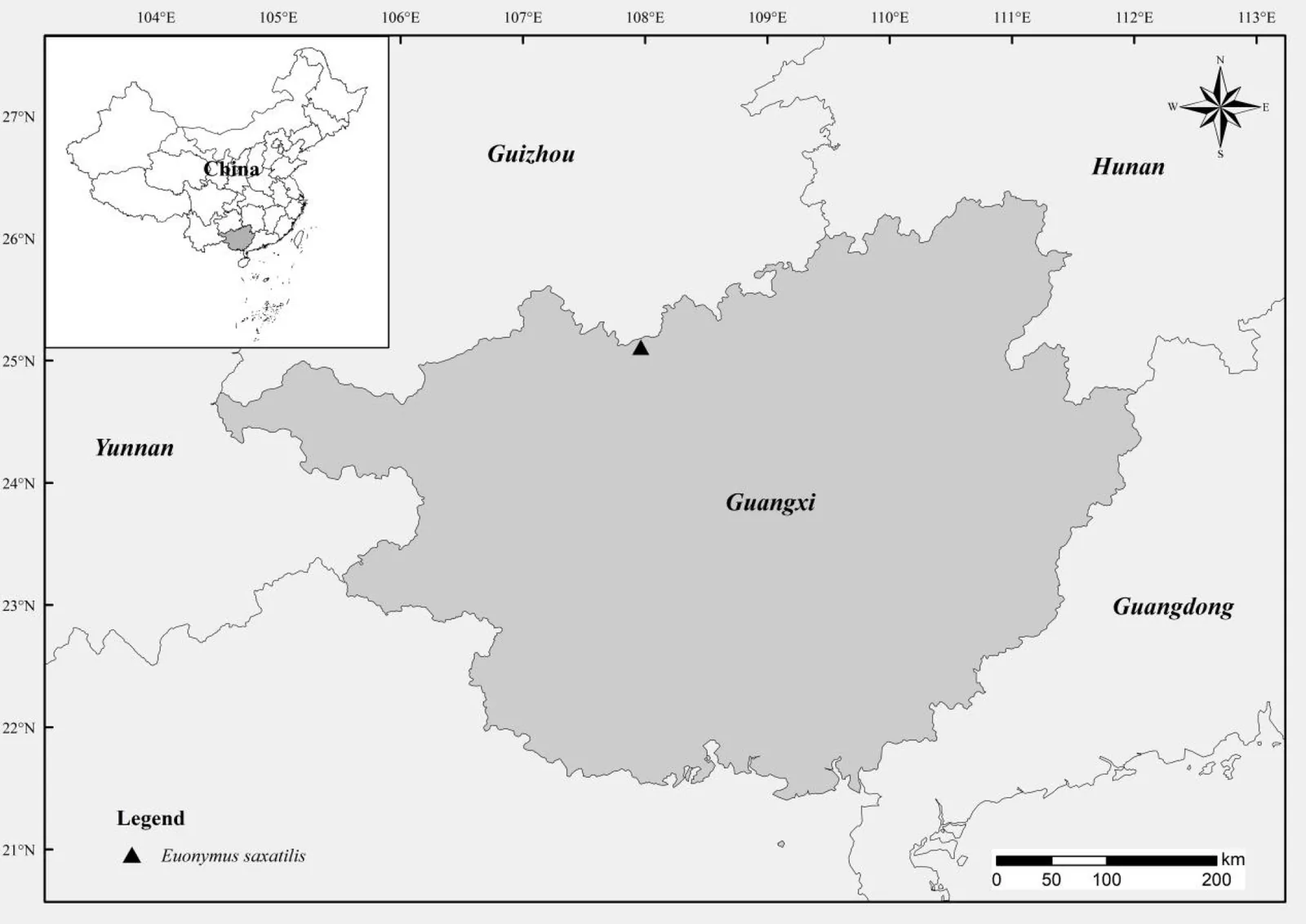
Distribution map of *Euonymus saxatilis*.

*Euonymus saxatilis* was only discovered in Guangxi Mulun National Nature Reserve, Huanjiang Maonan Autonomous County, Guangxi Zhuang Autonomous Region, China. It mainly grows in the upper-middle part of karst mountain slopes at an elevation of ca. 750 m (Fig. 1).

**Figure 2.**
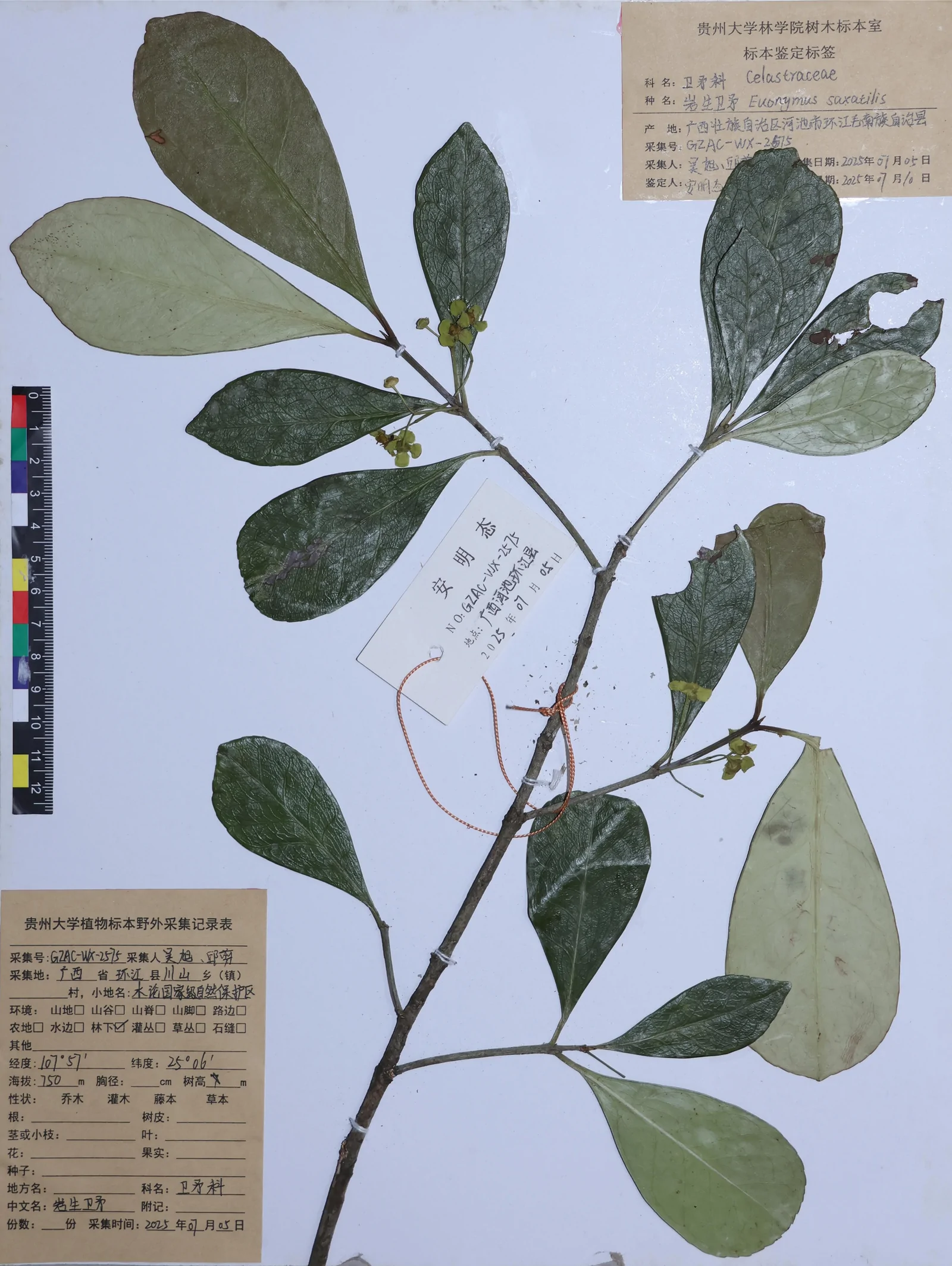
Holotype specimen of *Euonymus saxatilis*.

**Figure 3.**
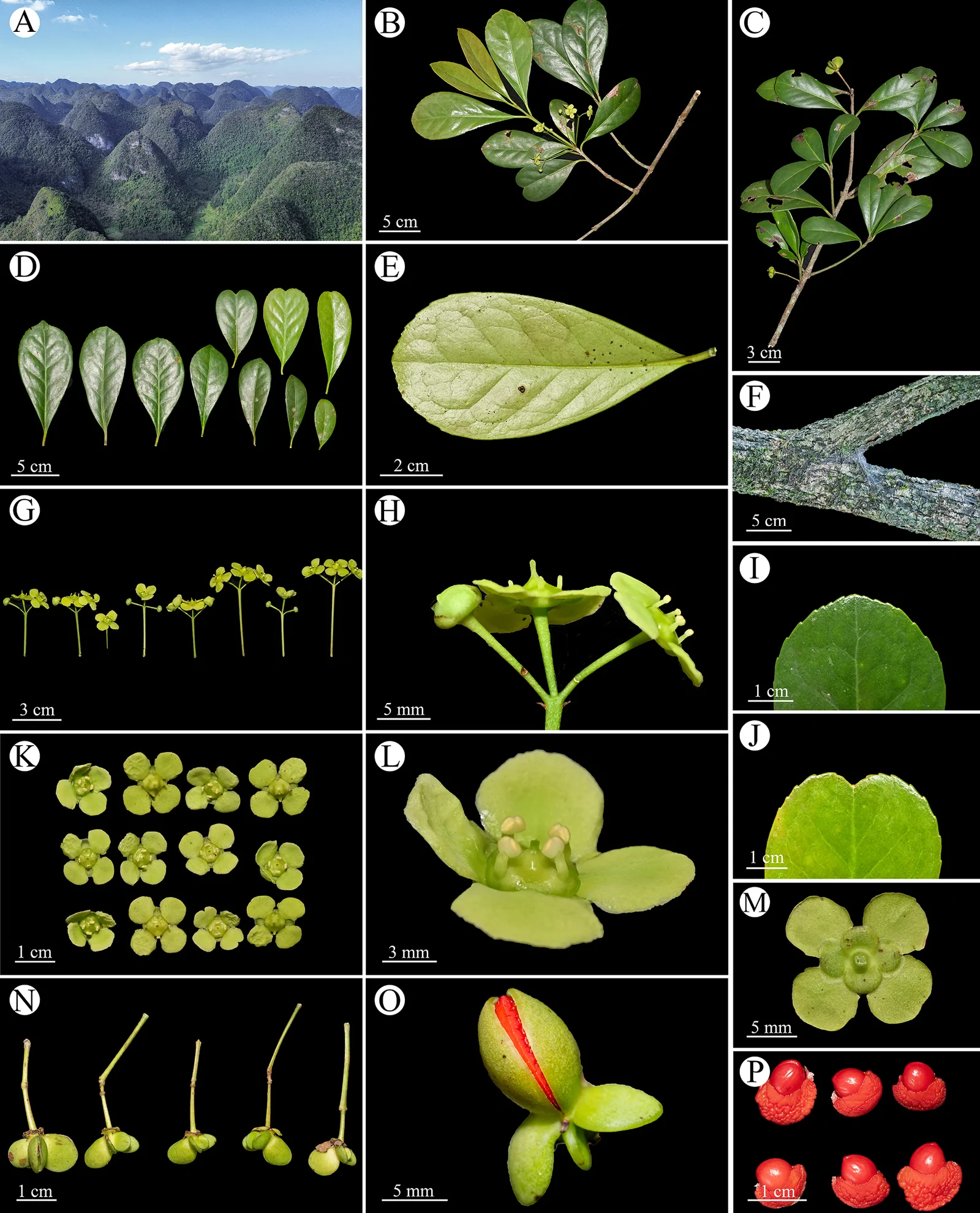
*Euonymus saxatilis*. **A**. Karst habitat; **B**. Branches with flowers; **C**. Branches with fruits; **D**. Adaxial leaf surface; **E**. Abaxial leaf surface; **F**. Stem; **G**. Inflorescences; **H**. Peduncle; **I, J**. Leaf apex; **K**. Flowers in front view; **L**. Flower in lateral view to show matured anthers; **M**. Flower in dorsal view; **N**. Infructescences; **O**. Cracked capsules; **P**. Seeds.

#### Conservation status.

*Euonymus saxatilis* is currently known from only a single wild occurrence locality, situated within Guangxi Mulun National Nature Reserve, Guangxi Zhuang Autonomous Region, China. It is exclusively found in habitats on the middle to upper sections of karst mountainsides at an elevation of approximately 750 m. There are approximately 20 individuals counted at this site. This species has an extremely narrow distribution range and a small population size, and is restricted solely to a specialized, narrowly endemic karst habitat. Therefore, the species is assessed as Critically Endangered (CR) according to the IUCN categories and criteria (IUCN 2022).

##### Phylogenetic position analysis

**Figure 4.**
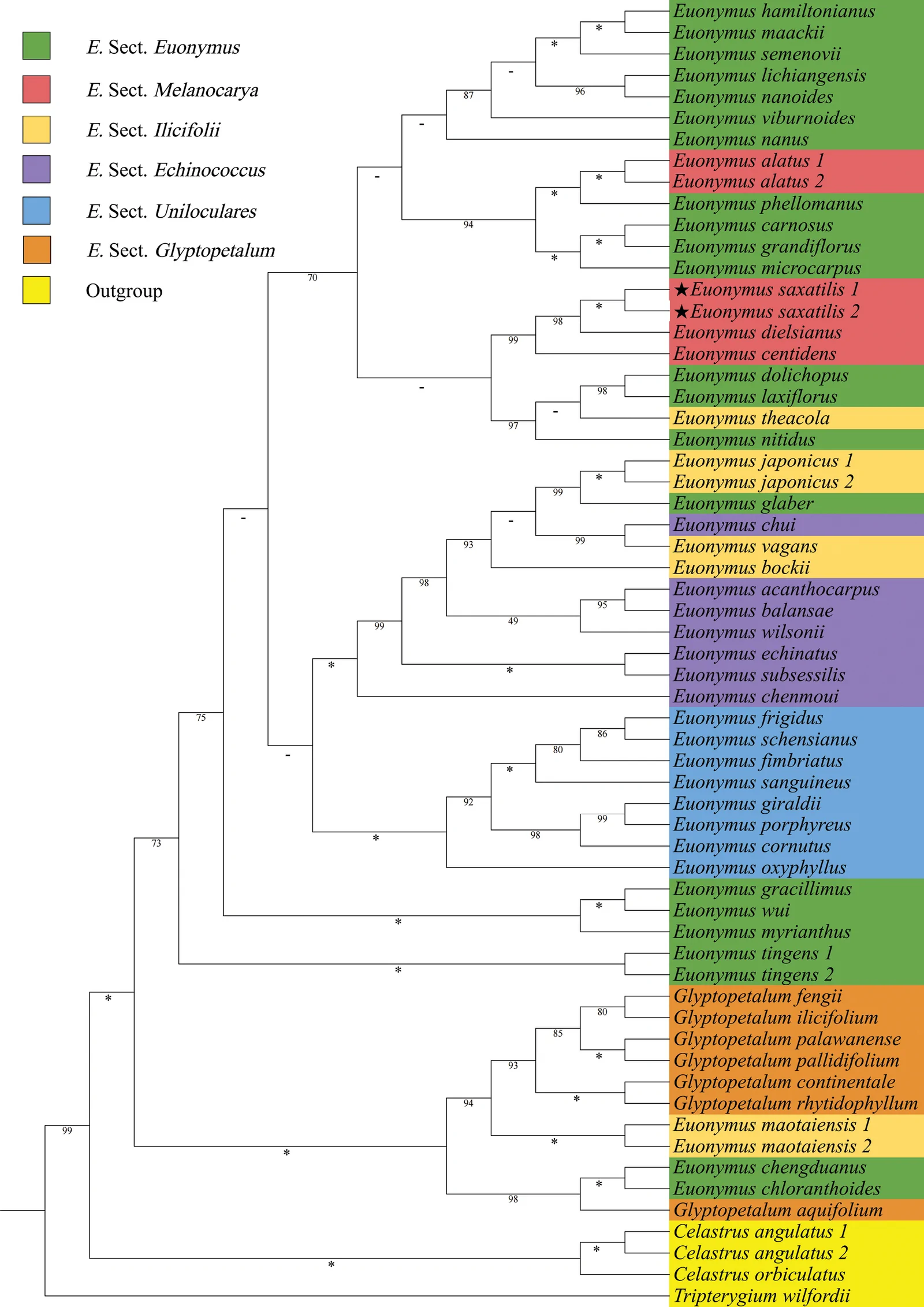
Maximum likelihood phylogenetic tree based on nuclear internal transcribed spacer (ITS) and *trnL-F* sequences of 61 accessions representing 55 taxa. Numbers at the nodes are maximum likelihood bootstrap values. Terminal nodes are in seven different colors corresponding to the five sections of *Euonymus* and *E.* sect *Glyptopetalum* described in Flora of China (Ma et al. 2008), as well as the outgroup. “black star” marks the *E. saxatilis*, “*”denotes 100% bootstrap support, “-”denotes a bootstrap support value below 70%.

Phylogenetic analyses based on the concatenated ITS and *trnL-F* sequences revealed that only species of *Euonymus* sect. *Uniloculares* formed a highly supported monophyletic clade (Fig. 4, BS = 100), whereas species from all other sections were intermixed across the tree. This topological pattern is largely congruent with the findings of previous studies (Hu et al. 2025). Furthermore, all accessions of *E. saxatilis* formed a monophyletic lineage with maximum nodal support (BS = 100), and were resolved as sister to *E. dielsianus* with strong bootstrap support (BS = 98). Collectively, these molecular phylogenetic results corroborate the recognition and taxonomic placement of the new species.

## Discussion

*Euonymus saxatilis* is readily distinguishable from *E. dielsianus* by a suite of key diagnostic traits, including leaf margin, leaf texture, peduncle length, and flower diameter. This species predominantly occurs on the middle to upper slopes of karst peak clusters. Its thick, coriaceous leaves represent a typical adaptive trait to the local arid habitats, as they can effectively reduce water transpiration. The relatively large leaf dimensions enhance light capture efficiency, making the species highly adapted to karst mountain environments characterized by high light availability and nutrient-poor conditions. Besides, the vast majority of Celastraceae species are primarily insect-pollinated. It is inferred that the larger floral structures and longer filaments of *E. saxatilis* may enhance its attractiveness to pollinators and improve pollen dispersal efficiency. Furthermore, integrating its core diagnostic morphological feature of deeply lobed capsules with its phylogenetic placement, we herein propose assigning this species to *E.* sect. *Melanocarya*.

## Supplementary Material

XML Treatment for Euonymus saxatilis

## Appendix 1

**Table A1.** The GenBank accessions for DNA sequences used in this paper.

**Species**	**GenBank Accession number**	
	**ITS**	***trnL–F***
*Celastrus angulatus* Maxim. 1	EU328759.1	JQ424262.1
*Celastrus angulatus* Maxim. 2	MH711208.1	JQ424261.1
*Celastrus orbiculatus* Thunb.	MN647936.1	MN655945.1
*Euonymus acanthocarpus* Franch.	KF282154.1	–
*Euonymus alatus* (Thunb.) Siebold 1	KF282156.1	EU328891.1
*Euonymus alatus* (Thunb.) Siebold 2	MH711819.1	EU328821.1
*Euonymus balansae* Sprague	KF282157.1	–
*Euonymus bockii* Loes. ex Diels	KF282158.1	–
*Euonymus carnosus* Hemsl.	KF282160.1	–
*Euonymus centidens* H. Lév.	KF282161.1	–
*Euonymus chengduanus* J. Hu & H. He	OR725028.1	–
*Euonymus chenmoui* W. C. Cheng	KF282162.1	–
*Euonymus chloranthoides* Yang	OR725029.1	–
*Euonymus chui* Handel-Mazzetti	KF282163.1	–
*Euonymus cornutus* Hemsl.	MH710947.1	JQ424310.1
*Euonymus dielsianus* Loes. & Diels	KF282167.1	–
*Euonymus dolichopus* Merr. ex J. S. Ma	KF282168.1	–
*Euonymus echinatus* Wall. ex Roxb.	KF282169.1	–
*Euonymus fimbriatus* Wall.	HQ393710.1	HQ393808.1
*Euonymus frigidus* Wall.	MW382667.1	–
*Euonymus giraldii* Loes. ex Diels	MW382662.1	–
*Euonymus glaber* Roxb.	HQ393703.1	HQ393812.1
*Euonymus gracillimus* Hemsl.	KF282173.1	–
*Euonymus grandiflorus* Wall.	HQ393711.1	HQ393789.1
*Euonymus hamiltonianus* Wall.	HQ393724.1	HQ393797.1
*Euonymus japonicus* Thunb. 1	HQ393700.1	AF534670.1
*Euonymus japonicus* Thunb. 2	KP214503.1	HQ393811.1
*Euonymus laxiflorus* Blume ex Miq.	KF282179.1	–
*Euonymus lichiangensis* W. W. Sm.	MH117525.1	–
*Euonymus maackii* Rupr.	MT923464.1	AF534669.1
*Euonymus maotaiensis* M.T.An & Xu Wu, sp. nov. 1	PZ539267	–
*Euonymus maotaiensis* M.T.An & Xu Wu, sp. nov. 2	PZ539268	–
*Euonymus microcarpus* (Oliv. ex Loes.) Sprague	KF282183.1	–
*Euonymus myrianthus* hemsl.	HQ393721.1	HQ393804.1
*Euonymus nanoides* Loes. & Rehder	KF282187.1	–
*Euonymus nanus* M. Bieb.	HQ393709.1	HQ393788.1
*Euonymus nitidus* Benth.	KP092574.1	JQ424311.1
*Euonymus oxyphyllus* Miq.	KF282190.1	HQ393810.1
*Euonymus phellomanus* Loes. ex Diels	HQ393718.1	HQ393791.1
*Euonymus porphyreus* Loes.	KF282196.1	–
*Euonymus sanguineus* Loes. ex Diels	MH808347.1	–
*Euonymus saxatilis* M.T.An , Jian Qiu & Xu Wu, sp. nov. 1	PZ539269	PZ533386
*Euonymus saxatilis* M.T.An , Jian Qiu & Xu Wu, sp. nov. 2	PZ539270	PZ533387
*Euonymus schensianus* Maxim.	MH710761.1	–
*Euonymus semenovii* Regel & Herder	KF282197.1	HQ393794.1
*Euonymus subsessilis* Sprague	KF282203.1	–
*Euonymus theacola* C. Y. Cheng ex T. L. Xu & Q. H. Chen	KF282204.1	–
*Euonymus tingens* Wall. 1	HQ393716.1	HQ393790.1
*Euonymus tingens* Wall. 2	MH117530.1	HQ393754.1
*Euonymus vagans* Wall.	HQ393720.1	HQ393809.1
*Euonymus viburnoides* Prain	KF282208.1	–
*Euonymus wilsonii* Sprague	KF282209.1	HQ393805.1
*Euonymus wui* J. S. Ma	JQ424145.1	JQ424312.1
*Glyptopetalum aquifolium* (Loes. & Rehder) C. Y. Cheng & Q. S. Ma	OK172405.1	–
*Glyptopetalum continentale* (Chun & F. C. How) C. Y. Cheng & Q. S. Ma	KF282210.1	–
*Glyptopetalum fengii* (Chun & F. C. How) Ding Hou	MZ568391.1	–
*Glyptopetalum ilicifolium* (Franch.) C. Y. Cheng & Q. S. Ma	OM985812.1	–
*Glyptopetalum palawanense* Merr.	HQ393705.1	–
*Glyptopetalum pallidifolium* (Hayata) Q.R.Liu & S.Y.Meng	KF282192.1	–
*Glyptopetalum rhytidophyllum* (Chun & F. C. How) C. Y. Cheng	HQ393706.1	JQ424313.1
*Tripterygium wilfordii* Hook. f.	HM115962.1	KY323585.1

## Additional information

### Artificial Intelligence (AI) use

The authors accept full responsibility for the content of the manuscript, including the disclosure of any use of AI.

No AI tools were used in the preparation of this manuscript.

### Author ORCIDs

J. Qiu https://orcid.org/0009-0006-7740-6270

X. Wu https://orcid.org/0009-0004-7935-6057

M.-T. An https://orcid.org/0000-0003-3886-0287

J.-H. Yu https://orcid.org/0000-0003-1765-8557

G.-L. Li https://orcid.org/0009-0009-5324-4643

### Data availability

All of the data that support the findings of this study are available in the main text or Supplementary Information.

## Supplementary materials

Supplementary material 1 Supplementary figure Data type doc https://binary.pensoft.net/file/1816604This dataset is made available under the Open Database License (http://opendatacommons.org/licenses/odbl/1.0/). The Open Database License (ODbL) is a license agreement intended to allow users to freely share, modify, and use this Dataset while maintaining this same freedom for others, provided that the original source and author(s) are credited. Jian Qiu, Xu Wu, Ming-Tai An, Jiang-Hong Yu, Guang-Lin Li

## References

[B1] Blume CL (1827) Bijdragen tot de flora van Nederlandsch Indië, pt. 17. Ter Lands Drukkerij, Batavia, 1146–1148. 10.5962/bhl.title.395

[B2] Capella-Gutierrez S, Silla-Martinez JM, Gabaldon T (2009) trimAl: a tool for automated alignment trimming in large-scale phylogenetic analyses. Bioinformatics 25(15): 1972–1973. 10.1093/bioinformatics/btp348 PMC2712344 19505945

[B3] Cheng JR, Ma JS, Huang PH (1999) *Euonymus*. In: Cheng JR, Huang PH (Еds) Flora Reipublicae Popularis Sinicae, vol. 45. Science Press, Beijing, 3–85.

[B4] Du C, Zuo YJ, Wu L, Liu QR, Ma JS (2015) Revision of five species of *Euonymus* (Celastraceae) from East and South Asia. Phytotaxa 213(3): 225–242. 10.11646/phytotaxa.213.3

[B5] Hou D (1963) Two additional Asiatic species of *Glyptopetalum* (Celastraceae). Blumea: Biodiversity, Evolution and Biogeography of Plants 12(1): 57–60.

[B6] Hu J, Huang X, Luo Y, Liu QR, He H, Liu Q (2025) *Euonymus chengduanus* (Celastraceae), a new species unexpectedly discovered in an urban forest park in the megacity of Chengdu, West China. Ecology and Evolution 15(4): e71234. 10.1002/ece3.71234 PMC11985356 40225884

[B7] Hu J, Zhang JY, Yu DX, Jiang H, Xu B, Qing L, He H (2022) *Euonymus aquifolium* (Celastraceae): Rediscovered in flowering with respect to its taxonomy, nomenclature, and rarity. PhytoKeys 201: 139–151. 10.3897/phytokeys.201.86180 PMC9848885 36762312

[B8] IUCN (2022) Guidelines for using the IUCN Red List categories and criteria. Version 15.1: Prepared by the Standards and Petitions Committee in July 2022.

[B9] Kalyaanamoorthy S, Minh BQ, Wong TKF, von Haeseler A, Jermiin LS (2017) Modelfinder: fast model selection for accurate phylogenetic estimates. Nature Methods 14(6): e587. 10.1038/nmeth.4285 PMC5453245 28481363

[B10] Katoh K, Standley DM (2013) Mafft Multiple Sequence Alignment Software Version 7: Improvements in performance and usability. Molecular Biology and Evolution 30(4): 772–780. 10.1093/molbev/mst010 PMC3603318 23329690

[B11] Li HY, Chen MD, Wang ZB, Hao ZY, Zhao XP, Zhu WY, Liu LC, Guo W (2022) Characterization of the Complete Chloroplast Genome and Phylogenetic Implications of *Euonymus microcarpus* (Oliv.) Sprague. Genes 13(12): e2352. 10.3390/genes13122352 PMC9778254 36553619

[B12] Liu HY, Meng SY, Liu QR (2021) A numerical taxonomic analysis of *Glyptopetalum* and *Euonymus* (Celastraceae). Guihaia 41(1): 92–102. 10.11931/guihaia.gxzw202004004

[B13] Liu L, Song TQ, Peng WX, Wang KL, Du H, Lu SY, Zeng FP (2012) Spatial heterogeneity of soil microbial biomass in Mulun National Nature Reserve in Karst area. Acta Ecologica Sinica 32(1): 207–214. 10.5846/stxb201011101618

[B14] Li X, Wang SL, Wu WH, Li M, Qin YH, Peng DR, Luo KW (2024) *Euonymus fangdingianus* (Celastraceae), a new cauliflorous species from southwestern China and northern Vietnam. Nordic Journal of Botany 2024(12): e04514. 10.1111/njb.04514

[B15] Li YN, Xie L, Li JY, Zhang ZX (2014) Phylogeny of *Euonymus* inferred from molecular and morphological data. Journal of Systematics and Evolution 52(2): 149–160. 10.1111/jse.12068

[B16] Loesener T (1902) Übersicht über die bis jetzt bekannten chinesischen Celastraceen. Botanische Jahrbücher für Systematik, Pflanzengeschichte und Pflanzengeographie 30: 446–474.

[B17] Ma JS (2001) A revision of *Euonymus* (Celastraceae). Thaiszia 11(2): 1–264.

[B18] Ma JS, Zhang ZX, Liu QR, Peng H, Funston AM (2008) Celastraceae. In: Wu ZY, Raven PH (Eds) Flora of China, vol. 11. Science Press, Beijing; Missouri Botanical Garden Press, St. Louis, 439–492.

[B19] Meng SY, Wang JL, Liu QR (2011) On the Identity of *Euonymus pallidifolia* (Celastraceae). Annales Botanici Fennici 48: 185–187.

[B20] Murray MG, Thompson WF (1980) Rapid isolation of high molecular weight plant DNA. Nucleic Acids Research 8(19): 4321–4326. 10.1093/nar/8.19.4321 PMC324241 7433111

[B21] Nguyen LT, Schmidt HA, von Haeseler A, Minh BQ (2015) IQ-TREE: a fast and effective stochastic algorithm for estimating maximum-likelihood phylogenies. Molecular Biology & Evolution 32(1): 268–274. 10.1093/molbev/msu300 PMC4271533 25371430

[B22] Qin YH, Bussmann RW, Li M, Liang YY, Wu WH, Yu SX (2025) *Euonymus duanensis* (Celastraceae), a new species from Guangxi, China. Nordic Journal of Botany 2025(10): e04828. 10.1002/njb.04828

[B23] Simmons MP, Mckenna MJ, Bacon CD, Yakobson K, Cappa JJ, Archer RH, Ford AJ (2012) Phylogeny of Celastraceae tribe Euonymeae inferred from morphological characters and nuclear and plastid gene. Molecular Phylogenetics and Evolution 62(1): 9–20. 10.1016/j.ympev.2011.08.022 22001302

[B24] Tan WN, Luo LJ, Nong SY, Huang XQ, Liu Y, Huang YS (2023) Primary study on species diversity of plant in Guangxi Mulun National Nature Reserve. Guihaia 43(12): 2182–2195. 10.11931/guihaia.gxzw202107051

[B25] White TJ, Bruns T, Lee S, Taylor J (1990) Amplification and direct sequencing of fungal ribosomal rna genes for phylogenetics. PCR Protocols 31: 315–322.

[B26] Wu X, Liu F, An MT, Yu JH, Yang WY (2026) *Euonymus maotaiensis* M.T.An & Xu Wu, sp. nov., a new species of *Euonymus* (Celastraceae) from southwest China. PhytoKeys 270: 143–153. 10.3897/phytokeys.270.171340 PMC12887579 41675601

[B27] Zhang D, Gao FL, Jakovlic I, Zou H, Zhang J, Li WX, Wang GT (2020) Phylosuite: An integrated and scalable desktop platform for streamlined molecular sequence data management and evolutionary phylogenetics studies. Molecular Ecology Resources 20(1): 348–355. 10.1111/1755-0998.13096 31599058

