# Supplementary material for: Euonymus saxatilis (Celastraceae), a new species from karst regions in Southwest China
Source: PhytoKeys. 2026 Oct 22;280:249–61. doi: 10.3897/phytokeys.280.195001 (PMC13720664; doi:10.3897/phytokeys.280.195001)
Supplement: Supplementary file 1 [file phytokeys-280-249_article-195001__-s001.doc]

**A** Holotype specimen of *Euonymus saxatilis;* **B** Holotype specimen of *Euonymus dielsianus；* **C** Holotype specimen of *Glyptopetalum fengii*
